# Importance of breastfeeding and complementary feeding for management and prevention of childhood diarrhoea in low- and middle-income countries

**DOI:** 10.7189/jogh.12.10011

**Published:** 2022-08-03

**Authors:** Davidson H Hamer, Hiwote Solomon, Gopika Das, Tanner Knabe, Jennifer Beard, Jon Simon, Yasir B Nisar, William B MacLeod

**Affiliations:** 1 Department of Global Health, Boston University School of Public Health, Boston, Massachusetts, USA; 2 Section of Infectious Diseases, Department of Medicine, Boston University School of Medicine, Boston, Massachusetts, USA; 3 Friedman School of Nutrition Science and Policy, Tufts University, Boston, Massachusetts, USA; 4 Doctor of Public Health Program, Boston University School of Public Health, Boston, Massachusetts, USA; 5 College of Engineering, Boston University, Massachusetts, USA; 6 Department of Maternal, Newborn, Child and Adolescent Health and Ageing, World Health Organization, Geneva, Switzerland

## Abstract

**Background:**

Early and exclusive breastfeeding have been shown to protect young infants from all-cause and diarrhoea-related mortality. Ideally breastfeeding should be initiated within the first hour of birth. Despite efforts to increase rates of early and exclusive breastfeeding in low- and middle-income countries (LMICs), challenges with uptake remain. This analysis reviews trends in early and exclusive breastfeeding, and the impact of infant feeding interventions in reducing childhood diarrhoea.

**Methods:**

We conducted a detailed review of articles written in English between 1990 and 2020 on the impact and efficacy of breastfeeding and complementary feeding on diarrhoea in children aged 0-2 years in LMICs. Using data from 86 countries and all WHO global regions collected from the mid-1980s through 2018 obtained from publicly available Demographic Health Surveys, we assessed trends in five-year intervals of timing of breastfeeding initiation, exclusive breastfeeding, median and mean duration of exclusive breastfeeding, and complementary feeding.

**Results:**

The literature search identified ten articles that described variable rates of early initiation of breastfeeding from 20% in Pakistan to 76% in Egypt. An analysis of 288 DHS studies found that the proportion of women who reported initiating breastfeeding within an hour of birth increased from 32% in the early 1990s to 55% between 2016 and 2020. Exclusive breastfeeding increased from 20% in the late 1980s to 48% between 2016 and 2020 and the mean duration of exclusive breastfeeding of 2-to-4-month-old infants doubled. Early initiation of breastfeeding and exclusive breastfeeding was associated with reductions in diarrhoea prevalence in the South East Asian, Western Pacific, Eastern Mediterranean, and African regions. Eight studies evaluating the effectiveness of different maternal education interventions, health care worker training, and media campaigns demonstrated improvements in exclusive breastfeeding, and most resulted in reductions in the incidence or duration of diarrhoea.

**Conclusions:**

During the last two decades, early and exclusive breastfeeding have increased. Nevertheless, the uptake of this basic, low-cost intervention remains suboptimal across all global regions. Given the potential benefits the in reduction of diarrhoea and diarrhoea-associated mortality, interventions for improving the uptake of early and exclusive breastfeeding in different sociological contexts need to be designed, implemented, and evaluated.

Breastfeeding has been consistently demonstrated to be protective against all-cause infant mortality and diarrhoea-related morbidity and mortality [1,2]. By increasing an infant's immune response to infectious pathogens, breastfeeding reduces the risk of severe illness and neonatal mortality. The potential impact is substantial, with a recent review suggesting that early and exclusive breastfeeding prevents half of all diarrhoea episodes in infants and 72% of hospital admissions [3]. Although complementary foods are beneficial for the growth and development of infants aged 6 to 12 months [4], they pose a potential risk of contamination during preparation, storage, and feeding.

Time to breastfeeding initiation is of paramount importance, with the World Health Organization (WHO) recommending initiation within the first hour of birth [5]. When breastfeeding is initiated early, the newborn ingests colostrum rich in immunoglobulins and other nutrients that confer protection against diarrhoea and other infectious diseases. Breastfeeding also provides direct skin-to-skin contact, important for prevention of neonatal hypothermia [6]. Delayed initiation of breastfeeding is associated with a higher risk of infant mortality and, despite varying data quality, increased risk of diarrhoea-related morbidity [7].

A recent analysis of time to initiation of breastfeeding in low- and middle-income countries (LMICs) using Demographic Health Survey (DHS) data revealed that the median time to initiate breastfeeding was about half an hour after birth [8]. However, the time to initiation of breastfeeding was greatly delayed in some countries in sub-Saharan Africa and South Asia, with Caesarean sections leading to delays in early breastfeeding. Immediate skin-to-skin contact was found to hasten time to initiation of breastfeeding.

The present analysis assesses factors contributing to low uptake of exclusive breastfeeding in LMICs, trends in early and exclusive breastfeeding based on DHS data, and the success rate of various infant feeding interventions in reducing childhood diarrhoea. This analysis will also assess the impact, uptake, facilitators, and barriers to exclusive breastfeeding and complementary feeding.

## METHODS

We conducted an extensive literature search in PubMed to identify relevant articles. Our search strategy included numerous variants of the following MeSH terms: “diarrhea,” “child,” “infant,” “breastfeeding,” “bottle feeding,” and “infant food.” The eligibility criteria for this analysis were established prior to the PubMed search. Inclusion criteria included articles written in English between 1990-2020 focused on the impact and efficacy of breastfeeding and complementary feeding on diarrhoea in children aged 0-2 years in LMICs. Studies were limited to observational studies, cohort studies, randomized controlled trials, experimental studies, and quasi-experimental studies. We excluded all studies which focused on special subgroups such as low birthweight or preterm babies, studies which did not directly assess the impact of breastfeeding and complementary feeding on childhood diarrhoea, and multi-intervention studies in which the impact or efficacy of feeding practices could not be independently extracted and analysed.

Each reviewer (TK, GD) independently assessed articles for eligibility. Disagreements on the inclusion of an article were resolved through discussion by the two reviewers. Quantitative and qualitative data were extracted and systematically entered into tables for further analysis.

In addition, DHS data on breastfeeding were extracted from Stat Compiler [9]. We analysed data from 288 surveys representing 86 countries and all WHO global regions collected from the mid-1980s through 2018. We analysed the following variables for infants aged 0 to 5 months: proportion of newborns who initiated breastfeeding within one hour of birth, exclusive breastfeeding, median and mean duration of exclusive breastfeeding, and complementary feeding. Standard WHO and DHS definitions were used with some minor modifications [10,11] (**Table 1**).

**Table 1:** Breastfeeding and complementary feeding definitions*

Variable	Definition
Ever breastfed	Percentage of children born in the last 24 months who were ever breastfed
Early initiation of breastfeeding	Percentage of children born in the last 24 months who were put to the breast within one hour of birth
Exclusive breastfeeding (under six months)	Percentage of infants 0-5 months of age who were fed exclusively with breast milk during the previous day
Mixed feeding	Percentage of infants 0-5 months of age who were fed formula and/or animal milk in addition to breast milk during the previous day
Predominant breastfeeding†	Percentage of infants 0-5 months having breast milk as the main source of food, with other foods as supplement
Continued breastfeeding (12-23 months)	Percentage of children 12-23 months of age who were fed breast milk during the previous day

*Extracted from “Indicators for assessing infant and young child feeding practices. Definitions and measurement methods.” WHO 2021 [10].

†Specialized definition used by some researchers.

The primary outcome of interest was the prevalence and duration of diarrhoeal disease in children between the ages of 0-2 years in LMICs. Our secondary outcomes included barriers to increased uptake of exclusive breastfeeding practices, the proportion and timing of initiation of exclusive breastfeeding and complementary feeding, and severity of diarrhoeal disease in children under 2 years of age. We tested for differences in mean proportions of initiation of breastfeeding, exclusive breastfeeding, breast- and complimentary feeding (mixed feeding), and duration of breastfeeding over time by comparing five-year time periods to the 1991-1995 period using the Wilcoxon rank-sum test and adjusting the level of significance for multiple comparisons using proc multtest in SAS 9.4. We chose the 1991-1995 period as the baseline because it was the earliest period for which we had information for all the outcomes.

## RESULTS

### Exclusive breastfeeding

The literature search identified ten articles that fulfilled the search eligibility criteria. Early initiation of breastfeeding (within an hour of birth) varied from 20% in Pakistan to 76% in Egypt (**Table 2**) [12,13,15-21]. Exclusive breastfeeding was practiced by a little over half of mothers in most countries for which data were evaluated. We compared these data with 288 DHS studies done over the last three decades, which yielded several relevant findings (**Tables 3** and **4**). The proportion of women who reported initiating breastfeeding within an hour of birth increased from 32% in the early 1990s to 55% between 2016 and 2020 (**Table 3**). The proportion of mothers practicing exclusive breastfeeding increased from 20% in the late 1980s to 48% between 2016 and 2020. Additionally, the mean duration of exclusive breastfeeding doubled for infants two to four months old. Early initiation of breastfeeding (within an hour of birth) was higher in Africa and the Americas relative to other global regions. In contrast, exclusive breastfeeding was lower in these regions and highest in the Southeast Asian Region (SEAR), where women also reported exclusive breastfeeding for the longest duration (**Table 4**).

**Table 2:** Frequency of breastfeeding practices prior to interventions

Authors	Year	Location	Early Initiation	Exclusive	Predominant	Partial	Continued
Clemens et al. [12]	1999	Egypt	76%	37%	N/A	94%	N/A
Ogbo et al. [13]	2018	Tanzania	43.4%	48.6%	10.1%	N/A	89.6%
Hanieh et al. [14]	2015	Vietnam	N/A	33.3%	N/A	N/A	N/A
Ogbo et al. [15]	2017	Sub-Saharan Africa	44.2%	29.2%	31.1%	N/A	83.3%
Sheikh et al. [16]	2020	Bangladesh	50.8%	55.3%	N/A	N/A	96.4%
Dhami et al. [17]	2020	India	42.3%	55.1%	19.3%	N/A	86.4%
Dagnew et al. [18]	2016	Ethiopia	N/A	16.7%	N/A	58%	N/A
Getachew et al. [19]	2018	Ethiopia	N/A	24.3%	N/A	30.9%	20.3%
Raheem et al. [20]	2017	Maldives Republic	N/A	0%	29.7%	61%	N/A
Saeed et al. [21]	2020	Pakistan	20.2%	53.6%	N/A	N/A	N/A

**Table 3:** Trends in exclusive breastfeeding over time based on Demographic Health Survey analysis

Time period	Studies	Initiated breastfeeding (%, 95% CI)	Exclusive breastfeeding (%, 95% CI)	Breast and complimentary (mixed) feeding (%, 95% CI)	Duration of exclusive breastfeeding median, mo (95% CI)	Duration of exclusive breastfeeding mean, mo (95% CI)
Pre-1991	31	No data	19.8 (13.3, 26.3)	20.0 (14.8, 25.2)	0.8 (0.5, 1.1)	2.1 (1.7, 2.6)
1991-1995	35	31.9 (23.2, 40.7)	28.8 (20.8, 36.9)	22.7 (16.2, 29.2)	1.3 (0.9, 1.7)	2.5 (2.0, 3.0)
1996-2000	53	39.4 (34.3, 44.5)	28.3 (22.6, 34.0)	21.0 (17.6, 24.4)	1.2 (0.9, 1.5)	2.4 (2.1, 2.8)
2001-2005	42	47.9 (43.3, 52.6)*	36.8 (31.5, 42.2)	18.4 (15.3, 21.6)	1.6 (1.2, 1.9)	3.1 (2.7, 3.4)*
2006-2010	47	52.5 (48.5, 56.4)*	43.2 (37.7, 48.7)*	18.5 (15.5, 21.6)	2.0 (1.6, 2.4)*	3.4 (3.1, 3.7)*
2011-2015	56	49.3 (45.1, 53.5)*	42.4 (37.2, 47.6)*	15.9 (13.8, 18.1)	2.0 (1.6, 2.4)*	3.4 (3.1, 3.7)*
2016-2020	24	55.3 (48.7, 61.8)*	47.6 (41.5, 53.7)*	14.4 (11.6, 17.3)	2.4 (1.8, 2.9)*	4.0 (3.7, 4.4)*
Total	288					

CI – confidence interval, mo – months

**Table 4:** Trends in exclusive breastfeeding by WHO region based on Demographic Health Survey analysis

Region	Studies	Initiated breastfeeding (%, 95% CI)	Exclusive breastfeeding (%, 95% CI)	Breast and complimentary feeding (%, 95% CI)	Duration of exclusive breastfeeding median, mo (95% CI)	Duration of exclusive breastfeeding mean, mo (95% CI)
*African	146	49.3 (46.2, 52.3)	33.7 (29.9, 37.4)	20.3 (18.3, 22.3)	1.6 (1.3, 1.8)	2.9 (2.7, 3.1)
Americas	48	53.2 (49.3, 57.1)	35.0 (29.0, 41.1)	17.2 (13.5, 20.8)	1.6 (1.2, 2.0)	2.9 (2.5, 3.2)
Eastern Mediterranean	28	38.5 (32.3, 44.6)	35.2 (29.3, 41.2)	15.3 (12.6, 17.9)	1.4 (1.0, 1.7)	2.9 (2.5, 3.3)
European	22	43.0 (35.4, 50.7)	31.9 (26.1, 37.8)	15.8 (13.1, 18.4)	1.2 (0.9, 1.6)	2.5 (2.0, 3.0)
South-East Asia	31	39.2 (31.1, 47.3)	47.5 (42.3, 52.8)	19.4 (14.1, 24.7)	2.2 (1.8, 2.6)	3.8 (3.5, 4.2)
Western Pacific	13	45.5 (36.6, 54.4)	39.7 (26.7, 52.8)	15.3 (10.3, 20.4)	1.9 (0.9, 2.8)	3.1 (2.3, 3.9)

CI – confidence interval, mo – months

*P-value<0.05 in comparison to 1991-1995.

Of the ten studies that assessed impact on infant diarrhoea, early initiation of breastfeeding and exclusive breastfeeding were both associated with important reductions in diarrhoea (**Table 5**) [12-16,18-21]. Reductions in diarrhoea prevalence occurred in SEAR, Western Pacific Region (WPR), Eastern Mediterranean Region (EMR), and African Region.

**Table 5:** Association of breastfeeding practices and diarrhoea incidence or prevalence*

Authors	Location	Early initiation of breastfeeding, % reduction (95% CI)	Exclusive breastfeeding, % reduction (95% CI)	Predominant breastfeeding, % reduction (95% CI)	Mixed feeding, % reduction (95% CI)	Continued breastfeeding, % reduction (95% CI)
Clemens et al. [12]	Egypt	-26% (-44%, -2%)	-33% (-53%, -3%)	N/A	-28% (48%, 1%)	N/A
Ogbo et al. [13]	Tanzania	0% (-19%, 25%)	-69% (-84%, -41%)	-70% (-90%, -11%)	N/A	24% (-58%, 264%)
Hanieh et al. [14]	Vietnam	N/A	-63% (-85%, -12%)	N/A	N/A	N/A
Ogbo et al. [15]	Sub-Saharan Africa	-19% (-23%, -15%)	-50% (-57%, -43%)	5% (-8%, 21%)	N/A	27% (5%, 55%)
Sheikh et al. [16]	Bangladesh	-31% (-49%, -6%)	-8% (-67%, 153%)	N/A	N/A	-71% (-91%, -2%)
Dhami et al. [17]	India	-27% (-31%, -22%)	-36% (-43%, -28%)	34% (16%, 55%)	N/A	-9% (-26%, 12%)
Dagnew et al. [18]	Ethiopia	N/A	-57% (-82%, -2%)	N/A	N/A	N/A
Getachew et al. [19]	Ethiopia	N/A	-68% (-83%, -38%)	N/A	-38% (-44%, -9%)	N/A
Raheem et al. [20]	Maldives Republic	N/A	-46% (-82%, 59%)	N/A	69% (-66%, 73%)	N/A
Saeed et al.	Pakistan	44% (-11%, 131%)	-34% (-55%, -2%)	N/A	N/A	N/A

*All studies measured diarrhoea incidence except Getachew et al [19].

Eight studies evaluated different types of maternal educational interventions, health care worker training, and use of media to improve breastfeeding practices (**Table 6**) [22-29]. All interventions led to improvements in exclusive breastfeeding and most resulted in reductions in the incidence or duration of diarrhoea. One study also demonstrated that an intervention to improve exclusive breastfeeding resulted in reduced risk of diarrhoea-related hospitalizations in young infants [26].

**Table 6:** Effects of training and behaviour change interventions on exclusive breastfeeding practices and diarrhoea

Authors	Location	Intervention	Impact on exclusive breastfeeding	Other key indicators
Froozani et al. [22]	Iran	Exclusive breastfeeding (EBF) education	Intervention = 54%, control = 5.6%	Mean duration EBF longer and reduced mean number of days with diarrhoea among intervention infants
Haider et al. [23]	Bangladesh	Two-hour counselling at hospitalization (of partially breastfed infants with diarrhoea) and repeat counselling two weeks after discharge	75% EBF post-intervention	No effect in 25% due to insufficient breast milk production and relatives pushing for early infant feeding
Bhandari et al. [24]	India	Trained healthcare workers counselling mothers on EBF	EBF at three months Intervention = 79%, control >48%	Lower seven-day diarrhoea prevalence at three months (0.64, 0.44-0.95, *P* = 0.028) and six months (0.85, 0.72-0.99, *P* = 0·04) vs. control
Jerin et al. [25]	Bangladesh	EBF training after delivery with phone call follow ups	Improved EBF at less than one month (85%-89% pre- and post-intervention) and five months (42% and 71% pre- and post-intervention)	No impact on diarrhoea
Zivich et al. [26]	Congo	Baby Friendly Hospital Initiative Steps 1-9 vs. 1-10	EBF prevalence at one week, steps 1-10 group = 96%, steps 1-9 group = 93%, control = 68%	Steps 1-9 associated with decreased incidence reported diarrhoea (IRR = 0.72, 95% CI = 0.53-0.99); steps 1-10 associated with decreased hospitalization for diarrhoea (IRR = 0.14, 95% CI = 0.03-0.60)
Nuzhat et al. [27]	Bangladesh	Breastfeeding counselling in hospitalized infants with diarrhoea	0.1% EBF on arrival vs. 65% EBF on discharge	
Greenland et al. [28]	Zambia	Neighbourhood forums, roadshows, radio messages on EBF	EBF of infants aged 0-5 months, intervention = 60.9%, control = 50.5%	EBF infants aged 0-2 months: intervention: 79%, control = 67%. Intervention raised awareness of need for zinc in treating diarrhoea
Huang et al. [29]	China	Antenatal education on EBF, monthly phone follow-ups for four months	EBF at hospital discharge: intervention: 43%, control = 32%	EBF at four months: intervention = 70%, control = 46%

CI – confidence interval, OR – odds ratio, EBF – exclusive breastfeeding. IRR – incidence rate ratio, mo – months

### Complementary feeding

12 studies addressed breastfeeding and complementary feeding [12-14,16-21,30,31]. Studies that evaluated the impact of early breastfeeding vs. complementary feeding found reductions in the duration of diarrhoea or diarrhoea-related hospitalizations in Egypt [12], Tanzania [13], Ethiopia [19], Vietnam [14], Bangladesh [16], India [17], the Maldives [20], and Pakistan [21]. A small multi-country study of 4158 infants in West Africa found a higher likelihood of diarrhoea among infants introduced to solid food at three to five months of age [30]. A large, multi-country analysis of DHS data on 83 000 mother-infant dyads in sub-Saharan Africa conducted between 2010 and 2014 yielded several important findings [15]. Early initiation of breastfeeding (OR = 0.81, *P*<0.001) and exclusive breastfeeding (OR = 0.50, *P*<0.001) were associated with decreased risk of diarrhoea [15]. By contrast, risk for diarrhoea increased with complementary feeding (OR = 1.27, *P* = 0.012) and continued breastfeeding at one year (OR = 1.27, 95% CI = 1.05, 1.55).[15]

### Factors influencing breastfeeding and early complementary feeding practices

Four studies shed light on factors influencing breastfeeding and early complementary feeding [22,23,27,30]. A 1997 study in Bangladesh identified several major reasons: maternal perception of inadequate breastmilk production, domineering grandmothers insisting on early complementary feeding, husband’s advice, concerns about financial insecurity due to inability to continue breastfeeding while at work, and maternal unwillingness to breastfeed [23]. Maternal concerns about producing enough breastmilk were also present in Iran [22]. A more recent study in Bangladesh reports other barriers to early, exclusive breastfeeding, including having a severely underweight baby, maternal concerns about inadequate breastmilk, and a perception that the newborn was not sucking well [27]. In Pakistan, lack of exclusive breastfeeding was associated with an infant age of four to five months, lower levels of maternal education, higher wealth index, urban residence, and lack of antenatal care [21].

A multi-country study in West Africa looking at the early introduction of complementary foods found child age to be a significant predictor in Benin, Burkina Faso, Côte d’Ivoire, Guinea, Niger, and Senegal [30]. Delivery by a trained traditional birth attendant was associated with increased likelihood of early infant feeding in Mali, Niger, and Senegal [30]. Paternal unemployment and birth order were associated with early complementary feeding in Benin [30]. In Burkina Faso and Niger, if the mother had one or more antenatal clinics as opposed to none, early introduction of complementary feeding was less likely [30]. In addition, higher household wealth index (Côte d’Ivoire), younger maternal age (Mali), and being a working mother (Niger) were associated with increased odds of early infant feeding, while female sex of the infant and rural location (Senegal) were associated with decreased odds [30].

## DISCUSSION

Studies which looked at the association between exclusive breastfeeding and diarrhoea consistently demonstrated breastfeeding’s protective effects on diarrhoea in the first six months of life and adverse consequences associated with complementary feeding. This in-depth literature review and analysis of data reported from individual studies found relatively low rates of early initiation of breastfeeding (within an hour of birth) and exclusive breastfeeding in the first six months of life. By contrast, the analysis of DHS data showed encouraging trends over time for the overall early initiation of breastfeeding and exclusive breastfeeding. This analysis also revealed a progressive increase in the duration of exclusive breastfeeding.

Interventions to improve basic early life nutritional practices are urgently needed. The evidence described above consistently shows that early initiation of breastfeeding (within an hour of birth) and exclusive breastfeeding for the first six months of life are associated with reduced risk of diarrhoea. Likewise, initiating breastfeeding within an hour of birth reduced the risk of severe illness requiring hospitalization and death [7]. While the DHS analysis suggests gradual improvements in early and exclusive breastfeeding over the last decade, room for improvement remains. Older child age, lack of antenatal care, and delivery by traditional birth attendants were all associated with a failure to exclusively breastfeed. The latter two factors can be addressed by ensuring universal access to antenatal care and facility-based delivery. We found early improvements of numerous behaviour change interventions such as infant feeding behaviours. These included communication campaigns using local media [28], education of mothers at the time of hospital discharge [22,23,26], and enhanced education of health care workers to emphasize the importance of exclusive breastfeeding [24]. All these interventions successfully improved early infant feeding behaviours.

This analysis has a few limitations. Among the individual studies found in the peer-reviewed literature, we found a wide range of study designs, sample size, population sampling, and differences in outcome definitions. As a result of the different types of study designs and differences in how breastfeeding and early infant complementary feeding were described, we were unable to perform a formal systematic review. In contrast, the DHS analysis used data collected using a common study design, adequate population size, population-based sampling and consistent data collection methods and instruments. Additionally, we extracted effect measures from studies that utilized raw data or estimates that could not be easily combined for potential meta-analysis. Given differences in study location, sample size, and study design, our analysis of the association between early and exclusive breastfeeding and infant diarrhoea was likely underpowered. Finally, since the major focus was to evaluate the association between breastfeeding and early complementary feeding practices and infant diarrhoea, qualitative studies of factors influencing these practices were not included so only a few of the quantitative studies identified in the literature search had relevant information that could be used to address this question.

## CONCLUSIONS

Current WHO recommendations promote initiation of breastfeeding within an hour of birth, exclusive breastfeeding for the first six months of life, and initiation of complementary feedings from the age of six months while continuing breastfeeding through two years of life [5]. These recommendations are based on solid empirical evidence showing multiple benefits including reduction of the incidence of diarrhoea (as demonstrated in this review), lower all-cause mortality [7], increased child intelligence [3], and potentially reduced risk of future diabetes mellitus and overweight [3]. The uptake of this basic, low-cost intervention remains suboptimal across all global regions with LMICs. The DHS datasets offer promising evidence of improvements [1,2], but more needs to be done. Evaluations of interventions to improve the uptake of early and exclusive breastfeeding in different sociological contexts are urgently needed to ensure effective implementation of WHO guidelines. High-potential strategies include integrated behaviour change interventions involving education of mothers by health care workers ante- and post-partum, communication through radio and TV, and innovative use of social media and mobile phone technology to further improve early and exclusive breastfeeding. A longer term strategy is to increase the average level of maternal education given evidence from secondary analyses of DHS datasets from South Asia that found a strong association between maternal completion of secondary or higher education and early initiation of breastfeeding, exclusive breastfeeding, and several different aspects of appropriate complementary feeding practices [32].
